# Contemporary Aspects of Burn Care

**DOI:** 10.3390/medicina57040386

**Published:** 2021-04-16

**Authors:** Arij El Khatib, Marc G. Jeschke

**Affiliations:** 1 Unité des Grands Brûlés, University of Montreal Medical Centre Sanguinet, 1051, Rue Sanguinet, Montréal, QC H2X 0C1, Canada; 2 Department of Surgery, Division of Plastic Surgery, Department of Immunology, Ross Tilley Burn Centre-Sunnybrook Health Sciences Centre, Sunnybrook Research Institute, University of Toronto, 2075 Bayview Avenue, Rm D704, Toronto, ON M4N 3M5, Canada; marc.jeschke@sunnybrook.ca

**Keywords:** burn history, burn advancement, burn research

## Abstract

The past one hundred years have seen tremendous improvements in burn care, allowing for decreased morbidity and mortality of this pathology. The more prominent advancements occurred in the period spanning 1930–1980; notably burn resuscitation, early tangential excision, and use of topical antibiotic dressings; and are well documented in burn literature. This article explores the advancements of the past 40 years and the areas of burn management that are presently topics of active discussion and research.

## 1. Introduction

Attempts to treat burn injury are as old as man’s use of fire. The first depictions of burn injury and treatment have been found in cave drawings [1]. Documentation of treatment recommendations were found in ancient Egyptian writings, the Ebers and Smith papyri, dating over 1500 BC (Before Christ), describing several treatment options including incantations, breast milk, and topical applications of multiple agents including honey, resin, and fabric strips soaked in oil as dressings [1,2,3,4]. Chinese medicine in the 6th century BC advocated the use of tea leaf extract on burns [2,3], while Hippocrates in the 4th century BC advocated multiple treatment options for burns including pig fat-soaked dressings, vinegar-soaked dressings, and balms made of oak bark [1,2,3]. Roman writings in the first century AD (Anno Domini) by Celsus also suggest different topical agents including honey, vinegar, bran, and exposure to air [2,3]. Arabian physicians used ice-cold water as advocated by Rhazes in the ninth century AC [2].

In the 16th century, Ambrose Pare advocated deep burn excision [4]. The first documented classification of burns was coined by Guilhelmus Fabricus Hildanus in the 17th century and was revisited multiple times by different authors including Richter and Dupuytren [3]. The three-degree classification most often used nowadays was coined separately by Petit in 1812 and Boyer in 1814 [3,5]. Reverdin recognized the importance of skin grafting in excised burn wounds, the standard treatment used for most deep burns today, in the 19th century [4]. Scotland is credited with starting the practice of treating burn patients in specialized burn units, with Syme establishing the first burn unit in Edinburgh in 1843 [6]. 

The 20th century saw a major leap in burn care and major improvements in patient survival. Forty-percent total body surface area burns in adults carried a 50% mortality in the post-World War II era, while in the 1990s, 50% mortality occurred in patients with burns of 80% of total body surface area [4]. This is due to numerous advancements that took place in both burn care, notably burn resuscitation and early excision; and non-burn specific advancements such as discovery of antibiotics and the tremendous improvements in intensive care therapy.

In 1930, Underhill published an article reporting the need for fluid resuscitation in major burn patients after studying blister fluid in patients of the Rialto fire of 1921 [7]. These observations generated further research that culminated in fluid resuscitation formulae utilized to estimate appropriate volume of intravenous fluids to be given to specific body surface area burns. The first resuscitation formula based on body surface area was suggested by Harkins in 1942. Cope and Moore, studying the burn victims of the infamous Coconut Grove Fire in 1942, published their formula based on both extent of burn and patient weight [8]. Other authors followed suit proposing and modifying multiple formulae; culminating in the introduction the formula most widely used nowadays, the Parkland formula, described by Baxter and Shires in 1968 [9]. 

The World Wars, with unprecedented air warfare that resulted in mass burns contributed to development of burn reconstructive surgery, as these patients were treated in dedicated wards that developed the expertise of the treatment teams. Sir Archibald McIndoe was a pioneer in the field of burn reconstruction for his work on severely burned World War II pilots in the UK [10,11].

Penicillin was discovered in 1929 by Alexander Fleming, but first documented antibiotic use in burn patients was first in the 1940s [12]. Antibiotic therapy, both topical and systemic, became a staple of burn treatment in the 1960s after publication of a series of papers on pseudomonal burn wound sepsis by Mason and Walker, and the reduction of postburn mortality by Pruitt et al. after use of mafenide acetate cream on burns in the 1960s [13,14,15,16].

While the tenets of burn surgery, namely the need for deep burn excision, remained the same as described centuries prior; the 20th century saw a paradigm shift to favor early excision as opposed to the earlier recommendations to wait until eschar separation [17,18]. The technique of tangential excision, popularized by Janzekovic in the 1970s, along with improvement in supportive care, made it possible to acutely excise large burns, resulting in decreases in mortality and length of hospital stay [3,18,19,20,21,22,23,24,25].

Most accounts of burn history deal with events up to the consolidation of the method of tangential early excision. Bridging the narrative to contemporary times, this article will discuss the major concepts, developments, and concerns of burn care of the last 40 years. Namely, these are (1) the research on and understanding of hypermetabolism as the pathophysiologic mechanism underlying the corporal response to major burns, (2) description of over-resuscitation for major burns, (3) the continuing battle with burn wound sepsis, (4) tissue bioengineering endeavors to produce an ideal skin substitute, and (5) use of lasers for modulation of burn scars (6) emphasis on mental health (7) rehabilitation, and (8) study of long-term outcomes of burn care. 

## 2. Hypermetabolism

It has long been known that major burns are far from local events, having effects on multiple organ systems and lasting for long periods of time; however, the exact mechanisms of these phenomena have not been well understood. Originally described in the 1930s and supported by studies performed in the 1940s and 50s, the concept of hypermetabolism as the systemic response to burn injury has come into the center-stage of the understanding of burn injury within the last two decades, with intense research on its molecular basis and possible treatments [26,27,28,29,30,31,32]. Hypermetabolism is described as a conglomeration of cellular phenomena occurring in response to major trauma, caused by complex hormonal and inflammatory interplays and consisting of changes in glucose, protein, and fat metabolism [27,33,34,35,36]. 

Burn injury initially causes an ‘ebb phase’ of metabolism characterized by decreased organ function and tissue perfusion which lasts 1–3 days [26]. This is followed by the ‘flow phase’ which consists of increased inflammatory cytokine secretion, increased tissue perfusion, heightened adrenergic and glucocorticoid responses, and decreased levels of growth hormone [37]. This stage may last up to 2 years after the burn, and as can be inferred, results in increased oxygen and energy expenditure, and caloric requirement [26]. 

While the evolutionary role of hypermetabolism is to provide the body with substrates to regenerate and fight off insults, it has been recognized that the severity and longevity of this response in burn injury surpasses the bodily needs, becomes deleterious and is in itself a cause of significant morbidity and possibly mortality in burn patients. Glycolysis, proteolysis and lipolysis cause significant catabolism, loss of lean body mass, physiologic exhaustion, delays in wound healing, and immune system dysfunction [37,38,39,40]. 

Traditionally, the two ways of off-setting hypermetabolism in major burn patients is to surgically excise the burn, thereby removing the major source of inflammation; and to provide the patient with adequate nutrition to limit lean body mass wasting and further catabolism [27,39,41].

The most powerful intervention that can be implemented in a major burn patient to curb hypermetabolism and decrease morbidity and mortality is undoubtedly early burn excision and closure of the resulting wounds [27,33]. Historically, this has become possible in the past 4 decades not because of advancement in surgical technique, as excision of burn wounds had been practiced for centuries; but because of advancements in supportive care allowing for the performance of safer surgery. The two fields allowing for this are intensive care which has provided the treatment of infections, blood transfusions, mechanical ventilation, blood pressure support, and enhanced patient monitoring; and the field of bioengineering that has provided skin substitutes allowing options for wound closure.

It is imperative to provide burn patients with adequate nutrition, currently, nutritional support is started enterally as early as possible, usually on the day of injury [27,42]. Nutritional requirements are calculated based on patients’ resting energy expenditure; inadequate nutrition is associated with muscle wasting which in turn can lead to immune dysfunction, impaired wound healing, infections, and death; conversely, excess nutrition can lead to hyperglycemia and fatty infiltration of organs [27,42,43]. There is no consensus on the ideal nutrition for burn patients, and while research is actively being conducted to determine the optimal ratios of macro- and micronutrients, it is generally accepted that the majority of calories are to be obtained from carbohydrates with careful control of fatty acids to avoid organ infiltration and dysfunction. It is also standard practice to provide patients with micronutrients including Vitamins A, C, and E; as well as selenium, zinc, copper, and iron to offset oxidative stress, modulate immune function, and promote wound healing [44]. Studies on individual essential amino acids such as glutamine and alanine are also being carried out [27,42,44].

While effective in attenuating the hypermetabolic response, burn excision and adequate nutrition do not completely halt it [26], resulting in attempts to mitigate it using pharmacological agents, including the following:

(a) Insulin: Hyperglycemia has been implicated in multiple detrimental processes in burn patients, including delayed wound healing, infections, and increased mortality. Conversely, keeping a burn patient’s glucose controlled around the 130 mg/dL mark has been shown to decrease patient mortality and morbidity associated with sepsis and infections [37,45,46,47,48]. Insulin was one of the first agents studied to curb hypermetabolism by controlling hyperglycemia and overcoming insulin resistance that develops in hypermetabolic patients. Insulin has also been shown to downregulate inflammatory cytokines and contribute to improved wound healing [27,37]. A drawback of insulin therapy is that it necessitates rigorous blood glucose measurements to avoid hypoglycemia which may be serious, and potentially fatal in the intensive care setting [37,45].
(b) Metformin: A possible replacement for insulin therapy that is currently being investigated for effectiveness of glucose control and hypermetabolism attenuation is Metformin. Its advantages are the easier dosing and less need for monitoring, as it does not cause hypoglycemia. Metformin may cause lactic acidosis however and renal failure in rare cases [27,37,45].
(c) Propranolol: A non-specific b-adrenergic blocker, propranolol has been shown to decrease the hypermetabolic response due to its ability to block the sympathetic response, thereby decreasing cardiac workload, insulin resistance, and loss of lean body mass, among other beneficial effects [27,49,50].
(d) Recombinant Human Growth Hormone (rHGH): rHGH has been studied as an agent for treatment of hypermetabolism due to the findings that its levels are low in hypermetabolic patients. While it has shown favorable outcomes in pediatric populations, including increase in lean body mass, it is seldom used in adults due to a study that citing high rates of mortality and morbidity in adults with the use of rHGH [27,37,51].
(e) Oxandrolone: A synthetic testosterone analog that possesses only 5% of testos-terone’s virilizing effects. Being an anabolic hormone, it helps in maintenance of lean body mass and has been shown to shorten hospital stay in burned children [32,52].

## 3. Fluid Creep 

Fluid resuscitation is a cornerstone of burn treatment; its study and protocolization in the 20th century have led to significant increases in burn patient survival [4]. However, recognition of over-resuscitation, known as fluid creep, and its detrimental effects on patient course and outcomes have dominated the past 2 decades [53]. In 2000, Pruitt famously wrote about the pendulum of resuscitation swinging in the direction of over-resuscitation of acute burns with crystalloid solution and emphasized the need to reverse this phenomenon [54]. 

There are recognized patient conditions that require higher-than-normal resuscitation volumes. These include very large total body surface area (TBSA) burns, inhalation injury, electrical injury, delayed presentation of burned patient, and polytrauma [55,56]. Recent literature suggests that routine burn patients without the previously mentioned conditions are increasingly receiving volumes of resuscitation fluid in excess of those advocated by resuscitation calculations [55,56,57,58,59,60]. Adverse effects of fluid creep include increased extremity pressures that may require release in the form of escharotomies or fasciotomies, airway edema potentially requiring intubation, and abdominal compartment syndrome [55,56,57,58,59,61]. 

While the exact mechanisms of fluid creep have not been delineated, it most probably is a multifactorial phenomenon caused by a combination of the following factors:

(a) Carelessness: patients receiving large volumes as runs by first responders, directly on admission, and reluctance of tapering of high volume infusions for fear of causing renal failure [55,60].
(b) Larger burns: the resuscitation formulae were described when burn survival in patients with very large burns was rare. Therefore, adequacy of the formulas is studied best in moderate-sized burns, whereas fluid requirements for large and very large burns may go beyond what can accurately be predicted by resuscitation formulas [9,55].
(c) Opioid creep: opioid analgesia, which is much more frequently used now than in the past, causes decreases in blood pressure which is then counteracted with larger resuscitation fluid volumes [55,62].
(d) Goal-directed resuscitation: resuscitation to achieve certain urine outputs or base-deficit figures without regard for clinical fluid balances and edema. Studies suggest that certain goals such as base deficit require 24–48 h to normalize even in the setting of adequate resuscitation [55,63]. Interim readings before value normalization may however prompt over-resuscitation [57].
(e) Pure crystalloid: patients resuscitated with crystalloid solution only require higher fluid volumes than those resuscitated with colloid [56,64]. In fact, the earliest version of the Parkland formula included colloid addition in the second day of resuscitation [55,56]. Colloids fell out of favor due to a study by Goodwin et al. in 1983 that showed increased mortality in patients receiving albumin [65]. Newer studies fail to demonstrate increased mortality with the use of colloids but also do not demonstrate a survival benefit with their use [60,66,67,68].

## 4. Sepsis in Burns

Sepsis is described as organ damage in the context of a dysregulated systemic inflammatory response to an infectious agent [69]. It is a primary cause of mortality in intensive care units worldwide and is presently the leading cause of death in patients with severe burns [70]. Due to this, sepsis has been the topic of much recent study and discussion. Its definition and diagnostic criteria have been revised frequently in the past three decades, leading to some confusion about the interpretation of study results and the appropriateness of comparison of different therapeutic trials due to use of different defining parameters. The need for standardization of the definition of sepsis has been recognized as a step towards clarity in the clinical diagnosis and therapeutic results of the condition. 

Traditionally, sepsis was defined as evidence of an infection in addition to a systemic inflammatory response syndrome (SIRS) [71]; whereas SIRS was defined as two or more of the following: temperature > 38 °C or < 36 °C, heart rate > 90 beats per minute, respiratory rate > 20 breaths per minute or maintenance of PaCO_2_ < 32 mmHg, or white bloodcount > 12,000/mm^3^ or 4000/mm^3^ or left shift defined as > 10% bands [72].The latest widely agreed-upon definition is the Sepsis-3 definition developed by the Third International Consensus Definitions for Sepsis and Septic Shock (Sepsis-3) in 2016 [73,74,75]. Sepsis-3 defines sepsis in terms of Sequential Organ Failure Assessment (SOFA) variables which are: PaO_2_/FiO_2_ ratio, Glasgow Coma Scale, mean arterial pressure, vasopressor requirements, serum creatinine or urine output, bilirubin, and platelet count; or quick SOFAs (qSOFAs) which are altered mental status (Glasgow Coma Scale GCS < 13), systolic blood pressure ≤ 100 mmHg, and respiratory rate ≥ 22 [73].SOFA and qSOFA variables are essentially proxies for organ dysfunction, and sepsis is defined as 2 or more SOFA criteria, or documented infection in addition to 2 or more qSOFA criteria [73,74,75].

Burn patients are habitually excluded from sepsis trials due to the overlap of traditional systemic inflammatory response syndrome (SIRS) symptoms such as tachycardia, tachypnea, fever, and leukocytosis with the inflammatory and hypermetabolic reactions seen in response to major burn injury [71,76,77]; therefore, sepsis definitions used in the general patient population are not validated in the burn patient population [71,78,79]. The American Burn Association (ABA) has developed a burn-specific definition of sepsis in 2007 in response to this dilemma, with the following criteria: temperature > 39 °C or < 36.5 °C, progressive tachycardia > 110 beats per minute, progressive tachypnea > 25 breaths per minute, thrombocytopenia < 100,000/mcL, hyperglycemia in the absence of pre-existing diabetes mellitus, inability to continue enteral feedings > 24 h [76]. In addition, the ABA definition requires that a documented infection is identified by a positive culture, or pathologic tissue source, or clinical response to antimicrobials [76]. Several trials have compared the Sepsis-3 and ABA criteria for predicting sepsis in the burn population and found Sepsis-3 to be superior to the ABA criteria [78]. It must be noted that Sepsis-3 definition has come under scrutiny for not being sufficiently specific for sepsis in burn patients [80]. Therefore, the pursuit for a satisfactory definition of sepsis in burn patients is still ongoing.

Major burn patients differ from the general patient population in terms of sepsis in that they present with loss of the body’s skin barrier function, which predisposes them to sepsis for prolonged periods of time; this is exacerbated by the immune compromise that is frequently observed in the context of major burns [71]. Additionally, major burn patients often require mechanical ventilation, central venous, arterial, and urinary catheterization, all of which further increase infection risk. As a result, rigorous infection prevention and control measures are the norm in modern burn units in an attempt to reduce the likelihood of infection. These measures include screening for resistant organisms upon admission and discharge of patients, individual patient rooms, contact isolation measures, a strong emphasis on hygiene, daily antimicrobial dressings for burn wounds, monitoring of need and status of all invasive catheters, and careful antimicrobial stewardship [70].

Effort has been put into collecting and protocolizing evidence on sepsis management, with the result being the Surviving Sepsis Campaign (SSC) guidelines that have been developed and published periodically since 2004. These guidelines are presented as collections of treatment recommendations, or ‘bundles’, that should be implemented within specific time frames or in response to certain signs and symptoms [81,82,83]. Adherence to these ‘bundle’ interventions has shown a decrease in mortality rates of sepsis in the general population [69]. While the management recommendations indicated in the Surviving Sepsis Campaign are mostly not new, consisting notably of intravenous fluid resuscitation, administration of antimicrobial agents after taking cultures, vasopressor support to maintain a (mean arterial pressure) MAP ≥ 65, renal replacement, and glycemic control [83]. It is the timeliness of these interventions that is an important predictor of survival in sepsis, with evidence of increase in mortality for each hour antimicrobial therapy is delayed after the onset of hypotension [84].

Most of the criteria included in any definition of sepsis are clinical. The few laboratory measurements included (platelet count, bilirubin) are non-specific. The development of a sepsis-specific laboratory marker could greatly help in the prompt diagnosis and follow-up of sepsis, especially in major burn patients, where most of the clinical signs and symptoms are common to both conditions and it is difficult to distinguish etiology. Currently, the two markers used as measures of sepsis are C-reactive protein (CRP) and Procalcitonin. While CRP is sensitive for inflammation, it is less specific for infection and is slower to change, with the half-life of several days [85]. The more expensive Procalcitonin is more specific to infection and has a shorter half-life and may therefore be useful as a marker of change in condition. Although it has been shown to be a relatively good marker for sepsis and survival in burn patients, its levels are subject to fluctuations in response to surgery and different types of microbial agents, making interpretations more complicated [77,86,87,88,89,90].

## 5. Skin Substitutes

Autograft donor sites in the body are limited. This limitation is compounded by the need for wound coverage after early excision, especially in the case of large burns [91]. One solution for this is the use of skin substitutes, which are naturally-occurring or manufactured alternatives to autografts that can be either temporary or permanent and replace the epidermis, the dermis, or both [17,92]. Ideally, a skin substitute should provide wound coverage to limit fluid loss and bacterial growth, reduce pain and allow for wound healing. There is no perfect skin substitute; the past 4 decades saw a boom of biotechnology in an attempt to make an ideal skin replacement. The following is a limited list of skin substitutes used in burn units today.

The earliest skin substitutes used were allografts, or cadaveric skin, first used by Girdner in 1881 [93,94]. Most major burn centers use allografts as a wound bed preparation material in moderate to large burns, to increase the likelihood of subsequent autograft take [93]. The drawbacks of allograft use are the need for resources (skin banks) for its storage, as well as its antigenicity which usually manifests at around 3 weeks after its application, necessitating its replacement with autograft.

One solution to the antigenicity and impermanence of allografts, while avoiding the morbidity and scarcity of traditional autografts is to culture skin from a small skin sample taken from the burn patient. Cultured keratinocytes, or cultured epithelial autografts (CEA), were first reported in 1981 by O’Connor et al. and required 3–5 weeks for the growth in vitro of sheets of epithelial cells from a small biopsy of a patient’s normal skin [17,95,96,97]. Advantages are lack of immunogenicity and negligible donor sites, disadvantages are fragility of the sheets (due to lack of dermal component which is what gives skin its elasticity and strength), high cost, and time for production [95,98,99,100]. CEA has evolved from culturing cells in sheets which may take up to 5 weeks, to culturing cells in suspension which takes 2–3 weeks [95,97]. Currently, several systems of suspended keratinocyte delivery are available in the market, these are usually applied over a dermal substitute in order to achieve some elasticity and strength [95,97].

Multiple attempts have also been made at producing a skin substitute that would simultaneously replace the epidermal and dermal layers, with the goal of achieving a skin substitute that is stronger, more elastic, and more resistant to wear than a cultured keratinocyte sheet. One such substitute is the self-assembled skin substitute (SASS) that is composed of a collagen-rich extracellular matrix produced by a patient’s fibroblasts which is then seeded with keratinocytes thereby producing a substitute that is non-immunogenic and contains both skin layers [91,98]. Limitations include the time needed for production and high cost.

Numerous non-human tissue skin substitutes have also been developed for use in the burn patient to replace the various skin components. While an exhaustive list of synthetic substitutes is beyond the scope of this text, three substitutes that are prevalent in modern burn units and warrant mention are Biobrane^®^, Integra^®^, and BTM^®^.

Biobrane^®^ is an epidermal substitute that is a synthetic bilayer consisting of an inner nylon mesh and an outer silastic membrane. It is most commonly applied to superficial second degree burns to act as a semi-occlusive dressing, thereby diminishing fluid loss and decreasing pain associated with dressing changes while the superficial burn heals spontaneously [101,102]. It has been particularly useful in pediatric patients with superficial second degree burns, but has also found uses in patients with non-burn epithelial defects such as toxic epidermal necrolysis syndrome (TENS) [103].

Integra^®^ is a dermal regeneration template developed in the 1970s by Yannis and Burke. It consists of a chondroitin-collagen dermis covered by a silastic epidermis [3,4]. The dermal matrix allows for migration of fibroblasts and macrophages and becomes vascularized and incorporated into the body, and the silastic epidermis is removed and autografted 3 weeks after application. Integra carries the advantage of easy storage and decreased contracture compared to autograft only [104,105], and of being able to survive on small exposed areas of bone or tendon, on which autograft alone does not survive. Disadvantages include infections and its high cost [17,105]. Biodegradable Temporizing Matrix, or BTM^®^, is a synthetic polyurethane dermal substitute developed in 2012 by Greenwood that incorporates into the body through ingrowth of blood vessels and fibroblast infiltration [106,107]. Like Integra^®^, it contains a sealing membrane that is removed 3–4 weeks after application, allowing the dermal matrix to be skin grafted [17,99]. Preliminary data show decreased contracture rates and decreased infections [99,100].

## 6. Lasers 

Burn scars are a well-recognized sequela of major burn injury. In addition to unsightly appearance, these can limit function through contracture formation, and cause neuropathic pain, itching, and repetitive wound breakdown [108,109,110,111,112,113]. Increased burn patient survival has meant an increased burden of burn scar morbidity and has brought the need for effective scar therapies to the forefront of burn care.

Traditionally, burn scar treatment has included conservative modalities such as compression garments, applications of intralesional steroids, silicone creams; and surgical modalities such as scar release or excision and grafting [108,109,110,111,112,113]. Laser therapy has emerged as a novel technique of manipulating scar tissue in the past 20 years [114].

Lasers can be classified as non-ablative and ablative, the difference being their mode of action. Non-ablative lasers target pigments within the skin, and may be used for hyperpigmentation, vascular anomalies and tattoo removal; while ablative lasers vaporize tissues, modulating scar tissue [111].

Pulse-dye lasers are an example of non-ablative lasers, they have a wavelength of 585 nm or 595 nm and target oxygenated hemoglobin within capillaries in the dermis causing the coagulation of these vessels resulting in decreased erythema in the scar [110,111,115,116,117,118]. Erbium-yttrium aluminium garnet (Erbium-YAG and CO2 lasers are examples of ablative lasers, they target abnormal collagen, destroying it and promoting formation of new collagen, consequently remodeling scar tissue [110,111,115,116,117,118]. Erbium-YAG has a wavelength of 2490 nm enabling it to target dermal matrix components, while the CO_2_ laser has a wavelength of 10,600 nm and is therefore able to effectuate more extensive tissue remodeling due to its ability to vaporize scar tissue and coagulate blood vessels in the scar at the same time. This higher energy however, also carries the potential for more complications due to the higher energy dispersal. Complications of laser therapy include erythema, swelling, pain, skin infection, and hyperpigmentation [110,111,115,116,117,118].

Extension of previous laser indications include laser therapy prior to definitive reconstructive surgery for contractures in an attempt to soften scar tissue, making it more malleable; as well as the topical application of corticosteroids just prior to laser therapy, the belief being that laser beams will allow enhanced delivery of the steroids into the scar [110,117,119].

## 7. Mental Health

The first time mental health was acknowledged as a major component of burn patient recovery was in the work of McIndoe on his patients who were mostly WWII soldiers [120]. The Guinea Pig Club was formed by his patients in 1941 to provide burn reconstruction patients with social and psychological support. Research done in the late 1980s and 1990s demonstrated that up to 45% of adult patients hospitalized for burn injury showed signs of post-traumatic stress disorder 1 year after their initial injury [121]. It has also been demonstrated that prevalence rates of psychological distress and anxiety are high in hospitalized patients and that these symptoms tend to persist after discharge [122]. Compounding the problem of psychiatric issues in burn patients is the high incidence of pre-existing psychiatric conditions, alcoholism, and substance abuse [123,124,125,126], which in some cases may be the inciting agents of the burn [127]. Patients with pre-existing psychiatric conditions have been found to have higher rates of complications and require longer hospitalizations after a burn injury, as well as more difficulties in rehabilitation and readjustment post-burn [128,129,130,131,132]. Acute stress disorder starts immediately after hospitalization and if left untreated may be a predictor of future post traumatic stress disorder (PTSD) [127,133].The burn team needs to be attuned to the patient’s psychological wellbeing and symptoms of stress, depression, anxiety, and sleep disturbance must be promptly recognized and treated. Mental health professionals such as counselors, psychologists and psychiatrists are an integral part of any burn unit.

Pain is strongly linked with stress, anxiety, and sleep disorders in burn patients; inversely, patients with these psychological symptoms also become less tolerant to pain and may even have decreased wound healing [134,135]. Pain is a strong predictor of both acute and long-term psychological sequelae, and both pain and psychiatric disorders are strong predictors of long-term functioning in burn patients [123,136,137,138,139]. Anxiety and depression caused by excessive pain are decreased with adequate pain management [135].

Pain management is of paramount importance to burn care. The ABA has published guidelines for the management of acute pain and the recommendations include the need for frequent burn assessments, pharmacological therapy that includes opioids as well as adjuncts such as acetaminophen and non-steroidal anti-inflammatory drugs (NSAIDs), agents for neurologic pain such as gabapentin and pregabalin, and the use of ketamine for procedural sedation when needed (by trained personnel) [140]. The guidelines also include the recommendation to offer patients nonpharmacological analgesia techniques, such as cognitive behavioral therapy, hypnosis and virtual reality (VR) when available. Hypnosis has been found to significantly reduce affective pain in burn patients as well as flashbacks to the inciting incident [141,142,143,144]. There is significant evidence for the use of virtual reality as a nonpharmacological analgesic technique, it has been found to decrease both pain and anxiety associated with dressing changes, procedures, and physiotherapy and is a powerful analgesic adjunct to pharmacological therapy [121,145,146,147,148,149,150,151,152]. Functional magnetic resonance imagine (MRI) imaging has shown decreased pain-related brain activity with the use of VR [153].

Burn patients need to reintegrate back into their lives and communities after discharge from the burn unit, this is a process that goes hand in hand with physical rehabilitation and may require education of the patients and their families as well as social support. Much in the same tradition of the Guinea Pig Club, burn survivor groups provide an understanding and supportive social network that can assist patients in their recovery [154,155]. Variables affecting success of reintegration include physical impairment, pre- and postburn psychological distress, and substance abuse, among others [122]. About 66% of adult patients are found to be working 2 years after their major burns [156], and presence of work correlates with a better subjective quality of life [157,158].

Followup of children who survive major burns reveals that most of them adapt satisfactorily [159]. In adolescents and young adults, 40–50% were found to be well-adjusted, 50–60% were found to have some degree of psychological distress, and 25% were found to have severe symptoms. In fact, the most debilitating long-term effects of childhood burns are psychological and not physical [160]. Social skills programs have been shown to improve psychosocial competence of adolescent burn survivors [159,160,161,162].

## 8. Rehabilitation

Burn treatment does not end with wound coverage or hospital discharge. Rehabilitation is an integral part of burn care, and probably the stage of burn management that lasts the longest. Burn injuries are notorious for function-limiting sequelae, including contractures, hypertrophic scarring, amputations, pruritus, thermoregulatory anomalies, hyperesthesias and paresthesias.

Rehabilitation has come to play a central role in burn management as survival of burn patients has improved, and quality of life and level of function of patients became the focus of recovery. It nowadays starts in the acute phase just after admission and carries on after patient discharge, and comprises of a vast variety of treatments including physiotherapy, ergotherapy, pain management, pressure garments, masks for hypertrophic scarring, and prosthetics [113,163].

The earliest rehabilitation intervention implemented after patient admission to the burn unit is positioning. The patient as well as the burned body part should be held in a position of comfort that nonetheless minimizes the chances of wound and joint contractures, edema, and pressure injury [164,165]. The strategies used for positioning may include techniques such as splinting, orthoses, special mattresses, foam cushions/wedges, and pressure dressings [165]. The particular strategies used are highly individualized and should be frequently reassessed and modified as the need arises.

The intermediate phase of rehabilitation spans the period of wound healing; priorities at this time are stretching of healing skin, grafts, and joints to prevent contractures and keep the tissues supple [166,167]. Long-term burn rehabilitation occurs after discharge and continues until the patient has gleaned the maximal benefit possible [166]. Rehabilitation programs range from inpatient to outpatient with frequent follow-ups, to patients becoming able to independently effectuate their programs with minimal oversight. The goals of long-term rehabilitation is to achieve maximal range of motion and functionality, and to learn to compensate for function that cannot be restored.

An important aspect of rehabilitation is scar management. Facial masks used to attenuate hypertrophic scarring of facial burns, as well as pressure garments used for scars for other body areas usually fall under the supervision of the burn physiotherapist. Evidence on the efficacy of pressure garments is not indisputable, with multiple studies falling on either side of the debate. The use of pressure to modulate scar healing was first mentioned in the medical literature in the late 1800s and was popularized in the 1970s; the supporters of garment use cite studies showing that application of pressure to a raised scar reduced its thickness and helped in its maturation [168,169,170,171,172,173,174]. The detractors argue that the garments do not apply adequate pressure, need to be worn for 23 h a day which is difficult to comply with; and that they cause discomfort, skin breakdown, and limitation of motion [175,176,177,178].

## 9. Long-Term Outcomes

Outcomes of burn care had historically been measured in terms of survival or length of stay. As survival rates of major burns increase, longer-term outcomes such as quality of life, psychosocial well-being, and return to work, may provide greater insights into the consequences of major burns. This in turn allows for patient-centric care and anticipation of patient needs not only in the immediate aftermath of a major burn, but also in the long-run [179,180,181]. Collection of long-term prospective data on multiple aspects of patient care and its results with respect to patient functioning, as well as periodic review of this data and identification of areas for improvement is a hallmark of major burn units. Verification by the American Burn Association stipulates that every verified burn unit collect data on burn admissions and their complications and outcomes, and creates a framework for collaboration of different burn units and teams on research.

## 10. Conclusions

Despite the impressive evolution that has taken place in burn care over the past century, there is ample room for further growth. The future will entail developments in the avenues discussed above. Dedicated efforts are being made in order to achieve better understanding and control of hypermetabolism, with ongoing trials on pharmacological agents to modulate the hypermetabolic response; glutamine and combination antihyperglycemics are currently being studied [27]. Research is also ongoing for biomarkers of hypermetabolism, with the goal of discovering markers that are easy to test for and that provide information on response to treatment and prognosis [27,182].

Likewise, optimal ways to resuscitate major burn patients with minimal side effects is an ongoing field of study, one notable trial that is currently in progress is the Acute Burn ResUscitation Multicenter Prospective Trial (ABRUPT2), evaluating acute resuscitation of major burns with crystalloid alone versus crystalloid with the addition of 5% albumin at 8 h post burn [183].

In the field of sepsis, active areas of investigation are the testing of sensitive and specific biomarkers of sepsis, with research into leukocyte biomarkers which may replace the currently used CRP and Procalcitonin measurements [184]. Another important field in sepsis research is the development of techniques that allow the detection of bacterial or fungal DNA within the bloodstream within hours, precluding the need to wait for days for antimicrobial culture results [85]. In addition, investigations on ways to develop therapeutic agents/biotechnology to allow the removal of inflammatory factors and cytokines from the bloodstream, therefore curbing the dysregulated reaction to infection in septic patients is underway [85].

Mesenchymal stem cells (MSCs) have been found to enhance wound healing capabilities [185,186]. It has also been discovered that burnt tissue that is excised in the process of burn debridement contains active MSCs. Research is ongoing on extracting these MSCs from burnt tissue, and incorporating them into a 3D-printed skin substitute that will not use healthy skin as a donor, and will not be immunogenic as the MSCs will come from a patient’s own burnt, discarded tissue [187,188]. If successful, this may prove revolutionary as a skin substitute option.

Evaluation and refinement of laser techniques and study of scar modulation techniques are also active areas of research, as are mental health interventions, and burn rehabilitation techniques. In the light of the better understanding of the pathophysiology and treatment of burns and the active research being conducted on many aspects of burn care, the future holds hope of improving outcomes and alleviating the suffering of burn patients.

## Data Availability

Data sharing not applicable.
